# Regulation of Calcium Homeostasis by PIEZO1 Drives NETosis and Fibrosis in Bronchopulmonary Dysplasia

**DOI:** 10.1111/jcmm.71096

**Published:** 2026-03-25

**Authors:** Lei Cao, Yan Mao, Chenxia Juan, Zhi Long, Qian Wang

**Affiliations:** ^1^ Department of Orthopedics Trauma, Trauma Center, Shanghai General Hospital Shanghai Jiao Tong University School of Medicine Shanghai China; ^2^ Department of Pediatric, Jiangsu Province Hospital of Chinese Medicine Affiliated Hospital of Nanjing University of Chinese Medicine Nanjing Jiangsu China; ^3^ Department of Nephrology, Jiangsu Province Hospital of Chinese Medicine Affiliated Hospital of Nanjing University of Chinese Medicine Nanjing Jiangsu China; ^4^ Department of Pediatric, Shanghai General Hospital Shanghai Jiao Tong University School of Medicine Shanghai China

**Keywords:** Bronchopulmonary dysplasia, calcium homeostasis, fibrosis, neutrophil extracellular traps, PIEZO1

## Abstract

Bronchopulmonary dysplasia (BPD) is a chronic lung disease primarily affecting preterm infants, characterised by impaired alveolar development and persistent inflammation, particularly associated with ventilator‐induced lung injury due to mechanical ventilation. In this study, we performed an integrated bioinformatic analysis of multiple datasets (GSE108754 and GSE39840) and identified 203 differentially expressed genes (DEGs) between BPD and control samples. Functional enrichment analysis revealed significant involvement in cytokine‐mediated signalling, response to lipopolysaccharide and regulation of interferon‐beta production. Using machine learning algorithms (LASSO, SVM‐RFE and Random Forest), we identified three hub genes (IL6, TFRC and PIEZO1) with high diagnostic accuracy for BPD. Immune infiltration analysis indicated altered immune cell proportions in BPD, with PIEZO1 expression positively correlated with neutrophil infiltration. Experimental validation confirmed elevated NETosis markers (PADI4, MPO, dsDNA) in BPD patients and further demonstrated that PIEZO1 overexpression promotes NET formation via calcium overload, which was inhibited by verapamil. Additionally, using a co‐culture system, we showed that PIEZO1‐induced NETosis exacerbates pulmonary fibrosis in lung epithelial cells. These findings highlight PIEZO1 as a key regulator of NETosis in BPD and a promising therapeutic target for mitigating lung injury and fibrosis.

## 1 Introduction

Bronchopulmonary dysplasia (BPD) is a chronic lung disease predominantly affecting premature infants [1], characterised by impaired alveolar and vascular development [2], and often leading to long‐term respiratory morbidity and increased mortality [3]. It remains one of the most common complications of preterm birth, affecting approximately 45% of infants with a birth weight below 1000 g and imposes a substantial clinical and economic burden on global healthcare systems [4]. A key contributing factor to the development and exacerbation of BPD is iatrogenic lung injury caused by mechanical ventilation, which imposes abnormal mechanical forces on the immature lung tissue [5]. Despite advances in neonatal care, including protective ventilation strategies, effective therapeutic strategies to prevent or reverse BPD progression are still lacking, highlighting the urgent need to elucidate its underlying molecular mechanisms and identify novel therapeutic targets.

A key pathological feature of BPD is persistent pulmonary inflammation, which disrupts normal lung development and promotes fibrotic remodelling [6]. Emerging evidence indicates that neutrophil extracellular traps (NETs)—web‐like structures composed of DNA, histones and granule proteins released by activated neutrophils—contribute significantly to pulmonary injury and inflammatory amplification [7, 8]. NET formation (NETosis) is driven by molecules such as myeloperoxidase (MPO), neutrophil elastase (NE) and peptidylarginine deiminase 4 (PAD4), which facilitates chromatin decondensation and extracellular trap release [9, 10].

PIEZO1 is a gene closely related to the mechanically activated ion channel that plays critical roles in sensing mechanical forces and transducing them into biological signals [11]. It is widely expressed in lung epithelial cells, endothelial cells and immune cells, where it regulates processes including vascular development, epithelial barrier function and inflammatory responses [12, 13]. Given its sensitivity to mechanical stress, PIEZO1 represents a plausible mediator linking ventilator‐induced lung injury to dysregulated immune activation and NETosis in BPD. Recent studies have implicated PIEZO1 in several pulmonary diseases due to its role in mechanotransduction and inflammation [14], its function in neutrophil‐mediated immunity and NETosis remains largely unexplored.

Although PIEZO1 has been associated with mechanosensitive signalling and inflammatory pathways, its role in regulating NETosis and contributing to lung injury in BPD is not well understood. Our study investigates the impact of PIEZO1 on neutrophil extracellular trap formation and its contribution to aberrant lung development and inflammation in BPD. We propose that PIEZO1 activation by mechanical forces promotes NETosis through calcium influx and downstream pro‐inflammatory signalling, exacerbating pulmonary damage and impairing alveolarisation. This study aims to elucidate the interplay between PIEZO1, NET formation and chronic inflammation in BPD, providing new insights into potential therapeutic strategies for this devastating neonatal disease.

## 2 Methods and Materials

### 2.1 Microarray Data and Processing

GEO database microarray datasets, such as GSE108754, GSE39840 and GSE235562, were obtained and combined for analysis. GSE108754 and GSE39840 were merged for training, while GSE235562 served as the test set. The R (version 4.5.1) was used to translate the probe names in the datasets to gene names. The training dataset had 10 BPD cases and 11 control samples after integration. The data was averaged for repeated probes and processed using log2 for large values. Batch effects were corrected using the “sva” package [15]. Principal component analysis (PCA) plots were made using the “ggplot2” program to demonstrate batch effect correction [16].

### 2.2 Screening for Potential Differentially Expressed Genes (DEGs) Linked to NETs

The “limma” software was used to extract DEGs from the combined dataset of GSE108754 and GSE39840. The data was fitted to a linear model, and the inference was stabilised by applying empirical Bayes moderation to the standard errors using the “eBayes” function. The parameters of |log_2_FC| > 0.585 and adjusted *p*‐value < 0.05 were used to filter DEGs. Volcano plots and heatmaps of DEGs were made using the “ggplot2” and “pheatmap” programs, respectively. Machine learning analysis was used to identify feature genes for BPD. The “WGCNA” software was used to conduct WGCNA [17]. A scale‐free *R* ^2^ = 0.95 and a power of β = 3 were the soft‐threshold parameters that ensured a signed scale‐free co‐expression gene network. In all, 13 non‐grey modules were created. Out of these nongray modules, the blue modules with the highest absolute correlation values with the BPD were selected for further analysis. In addition to NETs‐related genes (Table S1) retrieved from the geneCards website, a Venn diagram produced by the “ggvenn” R tool was used to further filter possible DEGs [18]. The correlation, expression box plot and line chart were generated by the “cor”, the “boxplot” and the “ggplot2” functions in the R program to visualise expression patterns of identified candidate DEGs between BPD and control samples.

### 2.3 Identification of Hub DEGs for BPD

The “glmnet” package was used to build a LASSO model with parameter tweaking by 10‐fold cross‐validation [19]. The response type was set to binomial, and alpha was set to 1. The least error criterion was used to choose feature genes. A support vector machine‐recursive feature elimination (SVM‐RFE) model was compared using the average misjudgement rates of their 10‐fold cross‐validation using the “e1071” software tool [20]. SVM‐RFE, a novel machine learning technique, may order features based on recursion to avoid overfitting. The “randomForest” software, which determined the number of trees with the lowest error, was used to generate an RF model [21]. Hub DEGs for BPD were identified using the previously stated methods.

### 2.4 Verification of the Identified Hub DEGs

Receiver operating characteristic (ROC) curves were created using the “pROC” program in order to verify the correctness of the DEG. Performance measures such as area under the curve (AUC), specificity and classification sensitivity were evaluated [22]. Additionally, the AUC of the hub DEGs was validated in the external testing cohort, GSE235562.

### 2.5 Functional Analyses

The “limma” package was used to identify genes differentially expressed between BPD samples and control ones, with criteria set at |log_2_FC| > 1 and adjusted *p*‐value < 0.05. Using the “clusterProfiler” R package, studies of the Kyoto Encyclopedia of Genes and Genomes (KEGG) and Gene Ontology (GO) were performed to look into possible molecular processes of these genes in BPD.

Using the “clusterProfiler” and “enrichplot” packages, single‐gene Gene Set Enrichment Analysis (GSEA) was carried out, dividing data into groups with high and low expression of the hub DEGs according to median expression values. To investigate pathway variations, the “c2.cp.kegg.Hs.symbols” subset from the MSigDB database was subjected to Gene Set Variation Analysis (GSVA). The “limma” and “GSVA” programs were used to investigate the variations in pathways and biological functions across subtypes.

### 2.6 Correlation Analysis and Immune Cell Infiltration

Single‐sample gene set enrichment analysis (ssGSEA), an extension of the traditional GSEA strategy, is a widely used technique in bioinformatics research to investigate immune infiltration. It was constructed using the R programming language and was used to quantify the proportions of 28 distinct immune cell types in the combined dataset. Violin plots were utilised to show how the immune cell composition of BPD and healthy samples differed. Heatmaps were used to show the locations of immune cells in the two groups, as well as the connection between immune cells and hub DEGs. The relationship between invasive immune cells and hub genes was investigated using the R program. The findings of the study were shown graphically using “ggpubr” package.

### 2.7 Primary Neutrophils and Serum Collection

The neutrophils were separated from donor blood. An EDTA tube was used to collect blood after written informed consent. The EasySep Direct Human Neutrophil Isolation Kit (STEMCELL Technologies) was then used to extract the neutrophils and serum in compliance with the manufacturer's instructions. All preterm infants with gestational age (GA) ≤ 29 weeks and/or birth weight (BW) < 1500 g, who were admitted to the NICU and finally diagnosed as BPD between August 2024 and March 2025 in Shanghai General Hospital, were eligible for inclusion in the study. Infants with significant congenital abnormalities, cardiac diseases other than PDA or patent foramen ovale, persistent pulmonary hypertension, genetic syndromes, or immunosuppression were excluded. The sample collection was approved by the Shanghai General Hospital Institutional Review Board.

### 2.8 Cell Culture and Transfection

The Chinese Academy of Sciences Shanghai Institute for Cell Resource Center provided the HL‐60 cell line, which was cultivated in RPMI 1640 medium (Gibco, Life Technologies Corporation, Gaithersburg, MD, USA) supplemented with 20% fetal bovine serum (FBS). Through 1.3% dimethyl sulfoxide (DMSO) induction, HL‐60 cells may transform into terminally mature neutrophil‐like cells, which are then known as dHL‐60. Lipofectamine RNAi max (Invitrogen, Grand Island, NY, USA) was used to transfect cells with siRNA (RiboBio, Guangzhou, China), and Lipofectamine 3000 (Invitrogen, Grand Island, NY, USA) was used to transfect overexpression plasmids of specific genes (GENEWIZ, Suzhou, China). Following a 24‐h transfection period, serum‐free RPMI 1640 media was used to replace the cells. The cells and cell culture supernatant were collected for further testing after a 24‐h period.

### 2.9 RNA Isolation and Quantitative Real‐Time PCR (qPCR) Analysis

Thermo Fisher Scientific's TRIzol reagent (Waltham, MA, USA) was used to extract total RNA. Following the manufacturer's instructions, a PrimeScript RT reagent Kit with gDNA Eraser (Takara, Tokyo, Japan) was used to reverse transcribe 1 μg of extracted RNA into complementary deoxyribonucleic acid (cDNA) [23]. On a Bio‐Rad CFX96 PCR System, a qPCR reaction was carried out using the SYBR Green Master Mix (Takara, Tokyo, Japan). Target protein mRNA expression levels were compared to matched controls using the 2^−ΔΔCt^ technique.

### 2.10 Western Blotting

Using a protease inhibitor cocktail (Roche Diagnostics, Indiana, USA) and RIPA Lysis and Extraction Buffer (Thermo Fisher Scientific, Waltham, MA, USA), proteins were extracted. The proteins were transferred to PVDF membranes (Millipore, Billerica, MA, USA) after being separated on SDS‐PAGE. Following blocking, matching antibodies were used to incubate the membranes [24].

### 2.11 Detection of Double‐Stranded DNA (dsDNA) Concentration

The Quant‐iT PicoGreen dsDNA assay kit (Thermo Fisher Scientific, Waltham, MA, USA) was used according to the manufacturer's instructions. In brief, we prepared a 5‐point standard from 1 ng/mL to 1 μg/mL by serial dilutions. After loading 50 μL of standards or samples onto black 96‐well plates, each sample was mixed with 50 μL of the Quant‐iT PicoGreen reagent's working solution and allowed to sit at room temperature for 5 min without exposure to light. A fluorescence reader was used to measure the intensity of the fluorescence (emission at a wavelength of 485 nm).

### 2.12 Cell Viability

The cell viability was assessed using standard MTT assays. 24 h after co‐cultivation, 96‐well plates were seeded with Beas‐2B cells. To treat cells for 1 h, MTT solution was applied to the medium. After gently removing the media, 150 μL of dimethylsulfoxide (DMSO, Sigma‐Aldrich) was introduced to dissolve the formazan. A plate reader (Bio‐Rad Laboratories) was used to measure the absorbance at 570 nm ± 10. The relative value of the control group represents the cell viability index.

### 2.13 Intracellular Ca^2+^ Measurement

A Calcium Colorimetric Assay kit was used to measure the intracellular Ca^2+^ content (Beyotime, Shanghai, China). Briefly, lysate buffer was used to lyse the dHL‐60 cells. The 50 μL cell lysates were then mixed with 75 μL of chromogenic reagent and 75 μL of calcium assay buffer, and they were incubated for 10 min. Finally, the absorbance at 575 nm was measured using the 96‐well microplate reader.

### 2.14 Statistical Analysis

To guarantee repeatability, the experiments were carried out three times. For comparisons involving many conditions, the Kruskal–Wallis test (with Dunn's multiple comparison post‐test) and the non‐parametric Mann–Whitney test are used to examine the data, which are given as mean ± standard deviation (SD). *p*‐values < 0.05 were regarded as statistically significant. In the figures, *p* < 0.05 is denoted by (*), *p* < 0.01 by (**) and *p* < 0.005 by (***).

## 3 Results

### 3.1 The Candidate DEGs Between BPD and Control Group

**FIGURE 1:**
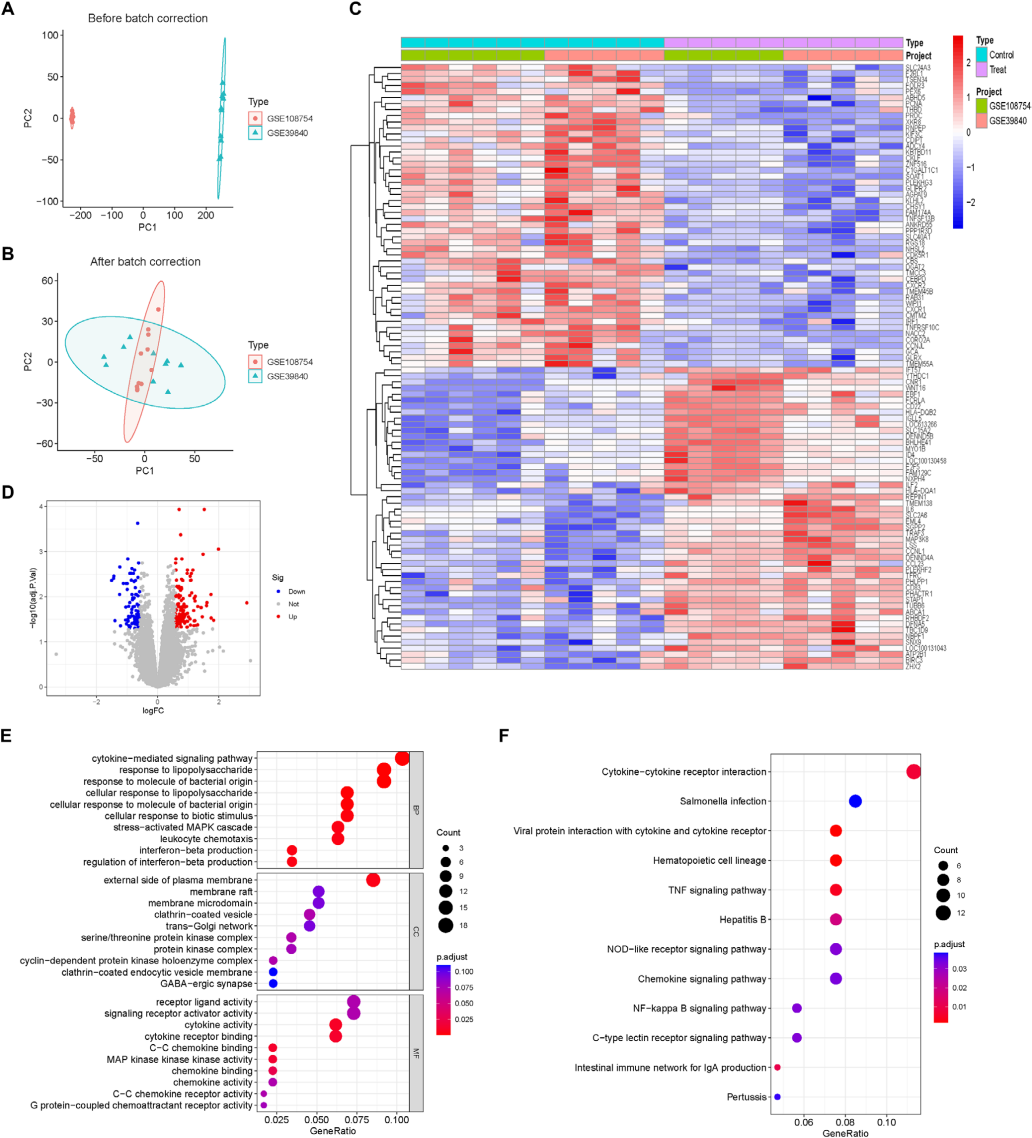
Differently expressed genes (DEGs) between BPD and control groups. (A) PCA diagram of three BPD‐associated datasets before calibration. (B) PCA diagram of these datasets after calibration. (C) Heatmap of DEGs. (D) Volcano plot of DEGs. (E) Histogram exhibited the results of the GO pathway analysis. (F) Histogram exhibited the results of the KEGG pathway analysis.

To reduce batch effects, the GSE108754 and GSE39840 datasets were combined and subjected to batch calibration. The efficiency of this calibration was evaluated using Principal Component Analysis (PCA; Figure 1A,B). A total of 203 DEGs were found by doing differential expression analysis on the combined dataset using the “limma” program to find DEGs between BPD and control samples. Figure 1C,D showed how the top 100 DEGs were expressed in the two groups. Functional pathway analysis was performed on genes that showed differences in expression between the BPD and control samples. This investigation showed participation in a number of biological processes, such as the cytokine‐mediated signalling pathway, response to lipopolysaccharide, stress‐activated MAPK cascade and regulation of interferon‐beta production. Regarding cellular components, the external side of the plasma membrane, membrane microdomain, clathrin‐coated vesicle and cyclin‐dependent protein kinase holoenzyme were identified. Molecular function analysis highlighted genes related to receptor ligand activity, cytokine activity, chemokine binding and signalling receptor activator activity (Figure 1E). Additionally, KEGG pathway enrichment identified cytokine‐cytokine interaction, haematopoietic cell lineage, NOD‐like receptor signalling pathway and NF‐kappa B signalling pathway (Figure 1F). These results indicated the potential functional enrichment pathways that may exist during the pathogenesis process of BPD.

**FIGURE 2:**
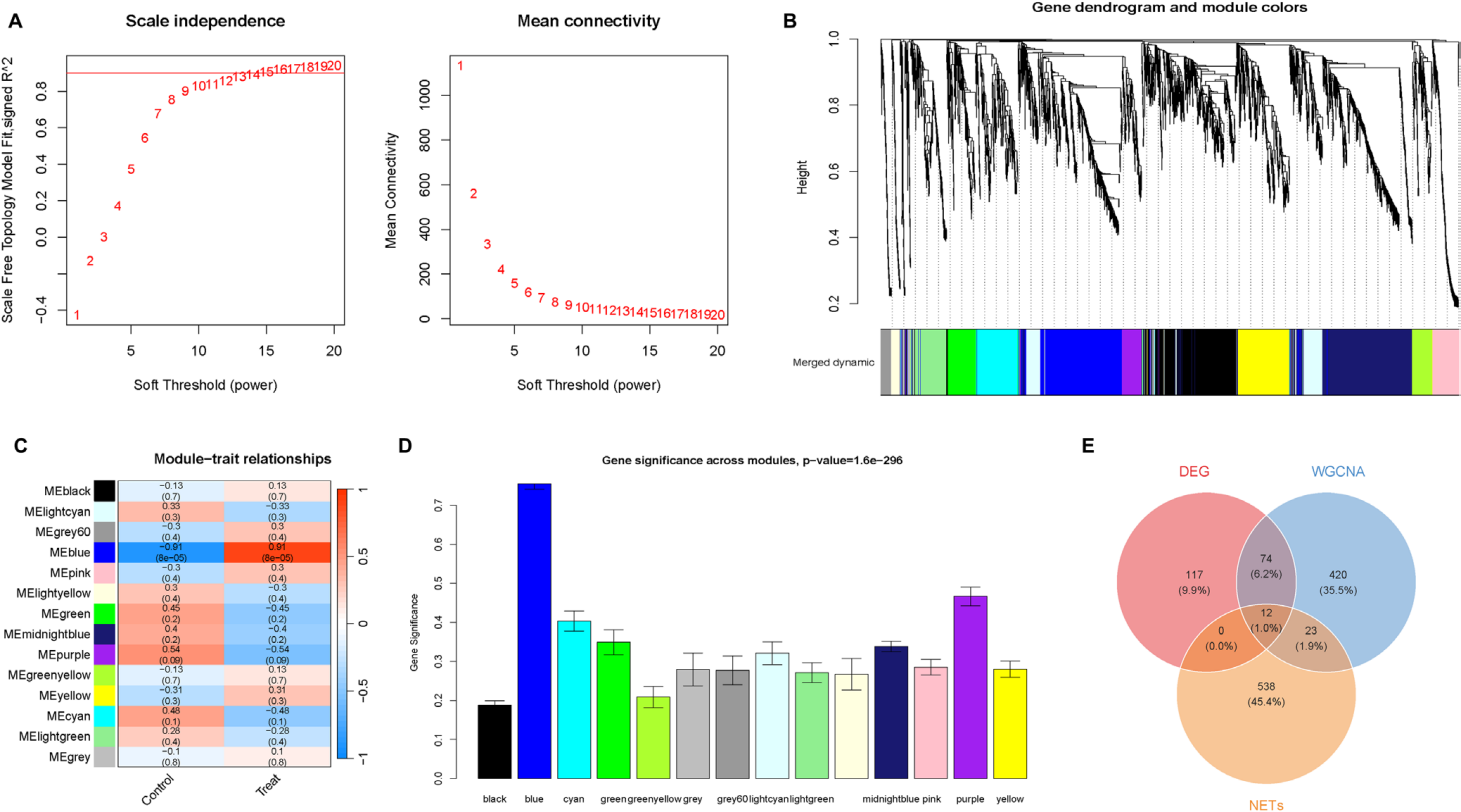
The candidate DEGs identified for NETs in BPD. (A–D) WGCNA analysis for BPD. (A) Optimal soft threshold selection. (B) Cluster dendrogram of WGCNA analysis. (C) Correlation between modules and occurrence of BPD. (D) The gene significance for the identified modules. (E) Venn plot exhibiting the reliable biomarkers among NETs, DEGs and WGCNA.

Next, we used the “WGCNA” R package to build the WGCNA network. According to the average linkage clustering, the expression patterns of the genes in the same module were comparable and significant. To guarantee a network free of scale, a soft threshold was established (Figure 2A). Modules marked in black, lightcyan, grey60, blue, pink, lightyellow, green, midnightblue, purple, greenyellow, yellow, cyan, lightgreen and grey were identified (Figure 2B–D). The result showed that the blue module was significantly associated with the BPD (Figure 2C,D). Combined with identified DEGs and NETs, which were obtained from the geneCards website, Venn analysis finally yielded 12 candidate DEGs simultaneously associated with them (Figure 2E). The 12 candidate DEGs were CXCR1, BIRC3, CXCR2, F2RL1, THBD, TFRC, IL6, PIEZO1, IRF1, NFKB1, TICAM1 and CSF3.

### 3.2 Verification of Candidate DEGs and Immune Cells Infiltration Analysis

**FIGURE 3:**
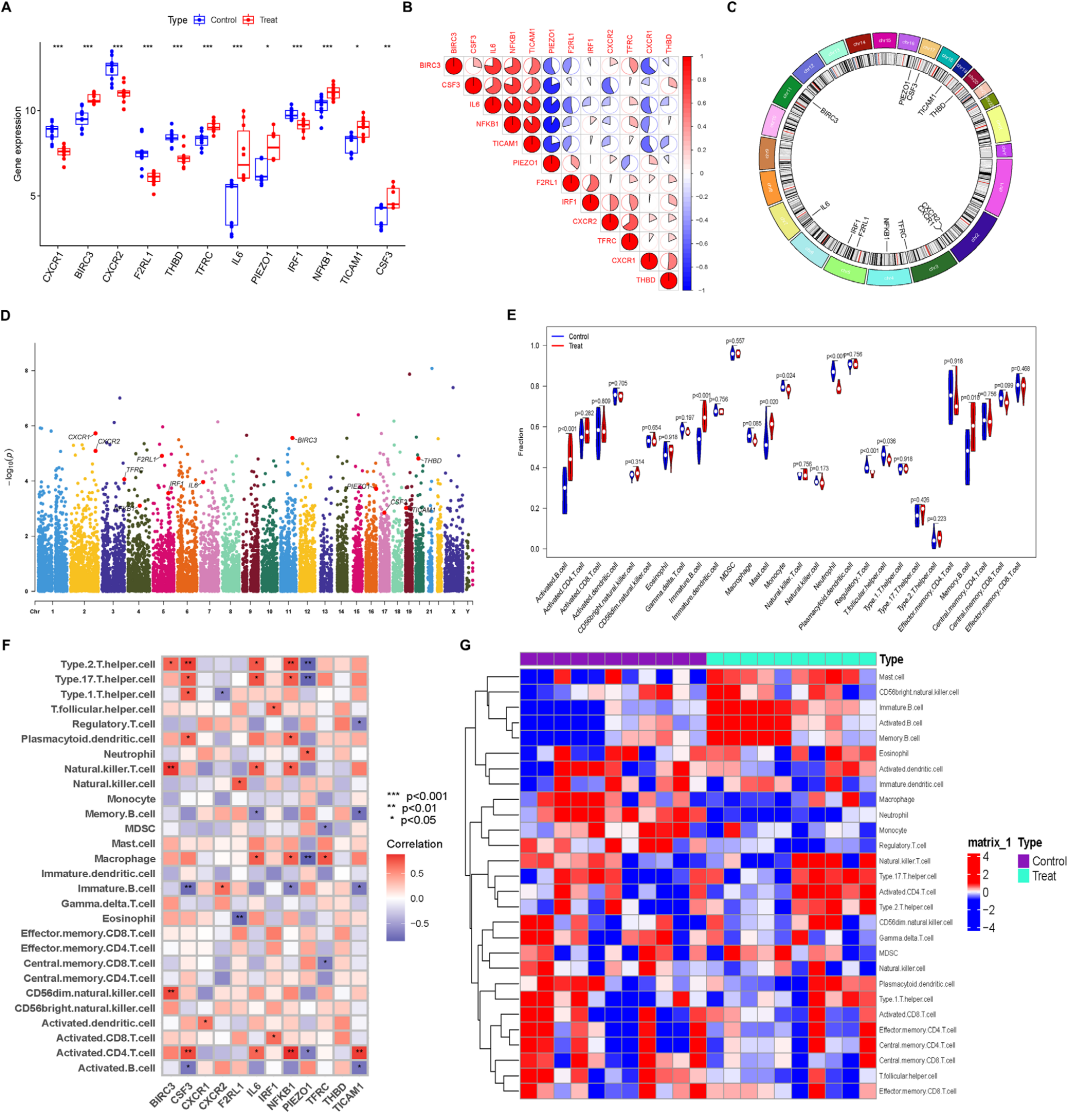
Expression of the identified candidate DEGs in BPD and further immune cells infiltration analysis. (A) Boxplot for the expression of 12 candidate DEGs in two groups. (B) Correlations between the candidate DEGs. (C) The location of the candidate DEGs on 23 chromosomes. (D) Manhattan plots ordered by chromosome position. (E) Difference in the immune cell infiltration of BPD and controls. (F) Correlations between the abundance of immune cells and the candidate DEGs. (G) Distribution of the immune cells of BPD and controls.

Compared with control samples, decreased CXCR1, CXCR2, F2RL1, THBD and IRF1 expression was observed in the BPD samples. Meanwhile, the expression of BIRC3, TFRC, IL6, PIEZO1, NFKB1, TICAM1 and CSF3 was higher in BPD samples than in control ones (Figure 3A). Figure 3B showed the correlation among 12 candidate DEGs. The location of the candidate DEGs on chromosomes was shown in Figure 3C. A Manhattan plot was completed to show chromosomal locations of associations with the candidate DEGs (Figure 3D).

The degree of immunological infiltration was assessed using the ssGSEA. Monocyte, neutrophil, regulatory T cell and T follicular helper cell infiltration levels were lower in the BPD group than in the control group. In the meantime, BPD was associated with an increase in activated B cell, immature B cell, mast cell and memory B cell (Figure 3E). Examination of correlation revealed the affirmative and negative correlations among candidate DEGs and immune cells (Figure 3F). Notably, PIEZO1 was positively correlated with Neutrophil. Heatmap in Figure 3G showed the distribution of immune cells in two groups, confirming the results of immunological infiltration in Figure 3E.

### 3.3 Identification and Verification of Hub DEGs

**FIGURE 4:**
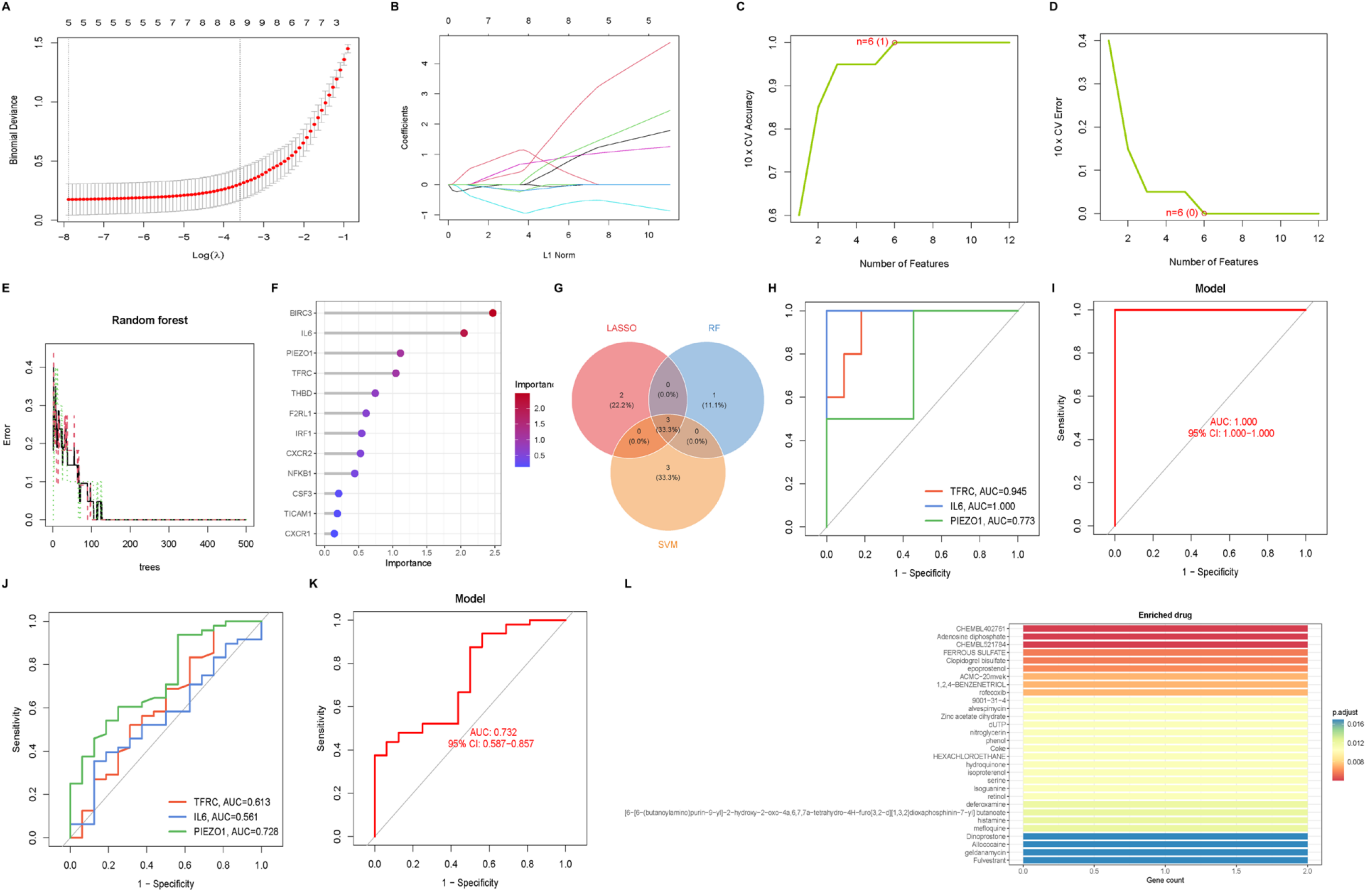
Identification and Verification of hub DEGs. (A, B) Regression coefficient path diagram and cross‐validation curves in LASSO logistic regression algorithm. (C, D) The curve of change in the predicted true and error value of each gene in SVM‐RFE algorithm. (E, F) The identification of feature importance based on random forests. (G) Venn diagram demonstrates the intersection of diagnostic markers obtained from the three algorithms. (H, I) ROC curves for evaluating the diagnostic ability of the hub DEGs. (J, K) ROC curves for evaluating the diagnostic ability of the hub DEGs in the external testing cohort, GSE235562. (L) Drug enrichment based on hub DEGs.

For a better understanding of the diagnostic potential of the candidate DEGs, we then constructed a prediction model for the diagnosis of BPD using three different algorithms to distinguish the BPD patients from healthy controls. Five out of twelve BPD‐related features of non‐zero coefficients were screened using the LASSO algorithm (Figure 4A,B). Six genes were then determined to be the best candidates for BPD based on SVM‐RFE after characteristics were chosen (Figure 4C,D). The top four genes were chosen as diagnostic genes after we used random forests to determine feature relevance (Figure 4E,F). Three hub genes (IL6, TFRC and PIEZO1) were identified for further actions when we finally intersected the candidate genes obtained from the SVM‐RFE, LASSO and RF models (Figure 4G). With AUC values of 0.945, 1.000 and 0.773 in the integrative cohort, respectively, the TFRC, IL6 and PIEZO1 demonstrated a significantly differentiating efficiency, according to the ROC curve, which was used to measure the predictive usefulness (Figure 4H). The AUC for the hub DEGs‐based model was 1.000 (Figure 4I), indicating the efficiency and accuracy of these genes in BPD. Interestingly, the results were validated in the GSE235562 cohort, and the consistent efficiency and accuracy for hub DEGs were obtained (Figure 4J,K).

To find possible medications that target hub genes, we searched the Enrich platform's DSigDB database. Table 1 showed the top 30 gene‐drug correlations. We extracted the top 30 chemical compounds based on *p*‐values and adjusted *p*‐values. The top 10 potential therapeutic compounds were chembl402761, adenosine diphosphate, chembl521784, ferrous sulfate, clopidogrel bisulfate, epoprostenol, ACMC‐20mvek, 1, 2, 4‐benzenetriol, rofecoxib and 9001‐31‐4 (Figure 4L).

**TABLE 1:** The details of top 30 predicted drugs based on the hub DEGs.

ID	Rich factor	Fold enrichment	*z* score	*p* value	*p* adjust	*q* value
CHEMBL402761	0.047619	311.8571	24.92211	1.34E‐05	0.004072	0.000129
Adenosine diphosphate	0.045455	297.6818	24.34662	1.47E‐05	0.004072	0.000129
CHEMBL521784	0.045455	297.6818	24.34662	1.47E‐05	0.004072	0.000129
FERROUS SULFATE	0.033333	218.3	20.83215	2.75E‐05	0.005706	0.00018
Clopidogrel bisulfate	0.029851	195.4925	19.70683	3.43E‐05	0.005706	0.00018
epoprostenol	0.026316	172.3421	18.49469	4.42E‐05	0.006128	0.000194
ACMC‐20mvek	0.02	130.98	16.10346	7.67E‐05	0.007233	0.000229
1,2,4‐BENZENETRIOL	0.019802	129.6832	16.02272	7.82E‐05	0.007233	0.000229
rofecoxib	0.019802	129.6832	16.02272	7.82E‐05	0.007233	0.000229
9001‐31‐4	0.014815	97.02222	13.83472	0.00014	0.010821	0.000342
alvespimycin	0.014493	94.91304	13.6814	0.000146	0.010821	0.000342
Zinc acetate dihydrate	0.013423	87.90604	13.15924	0.000171	0.010821	0.000342
dUTP	0.011173	73.17318	11.98738	0.000246	0.010821	0.000342
nitroglycerin	0.010753	70.41935	11.75539	0.000266	0.010821	0.000342
phenol	0.010753	70.41935	11.75539	0.000266	0.010821	0.000342
Coke	0.010526	68.93684	11.62859	0.000277	0.010821	0.000342
HEXACHLOROETHANE	0.010526	68.93684	11.62859	0.000277	0.010821	0.000342
hydroquinone	0.010471	68.57592	11.59751	0.00028	0.010821	0.000342
isoproterenol	0.010471	68.57592	11.59751	0.00028	0.010821	0.000342
serine	0.010471	68.57592	11.59751	0.00028	0.010821	0.000342
Isoguanine	0.010417	68.21875	11.56667	0.000283	0.010821	0.000342
retinol	0.010363	67.86528	11.53606	0.000286	0.010821	0.000342
deferoxamine	0.009615	62.97115	11.10368	0.000332	0.012021	0.00038
[6‐[6‐(butanoylamino)purin‐9‐yl]‐2‐hydroxy‐2‐oxo‐4a,6,7,7a‐tetrahydro‐4H‐furo[3,2‐d][1,3,2]dioxaphosphinin‐7‐yl] butanoate	0.009009	59	10.74006	0.000378	0.012221	0.000387
histamine	0.008969	58.73543	10.7154	0.000382	0.012221	0.000387
mefloquine	0.008969	58.73543	10.7154	0.000382	0.012221	0.000387
Dinoprostone	0.007092	46.44681	9.499526	0.00061	0.016954	0.000536
Allococaine	0.006897	45.16552	9.363668	0.000645	0.016954	0.000536
geldanamycin	0.00639	41.84665	9.002237	0.000751	0.016954	0.000536
Fulvestrant	0.00625	40.93125	8.899967	0.000785	0.016954	0.000536

### 3.4 Function Enrichment Analysis Based on the Hub DEGs

**FIGURE 5:**
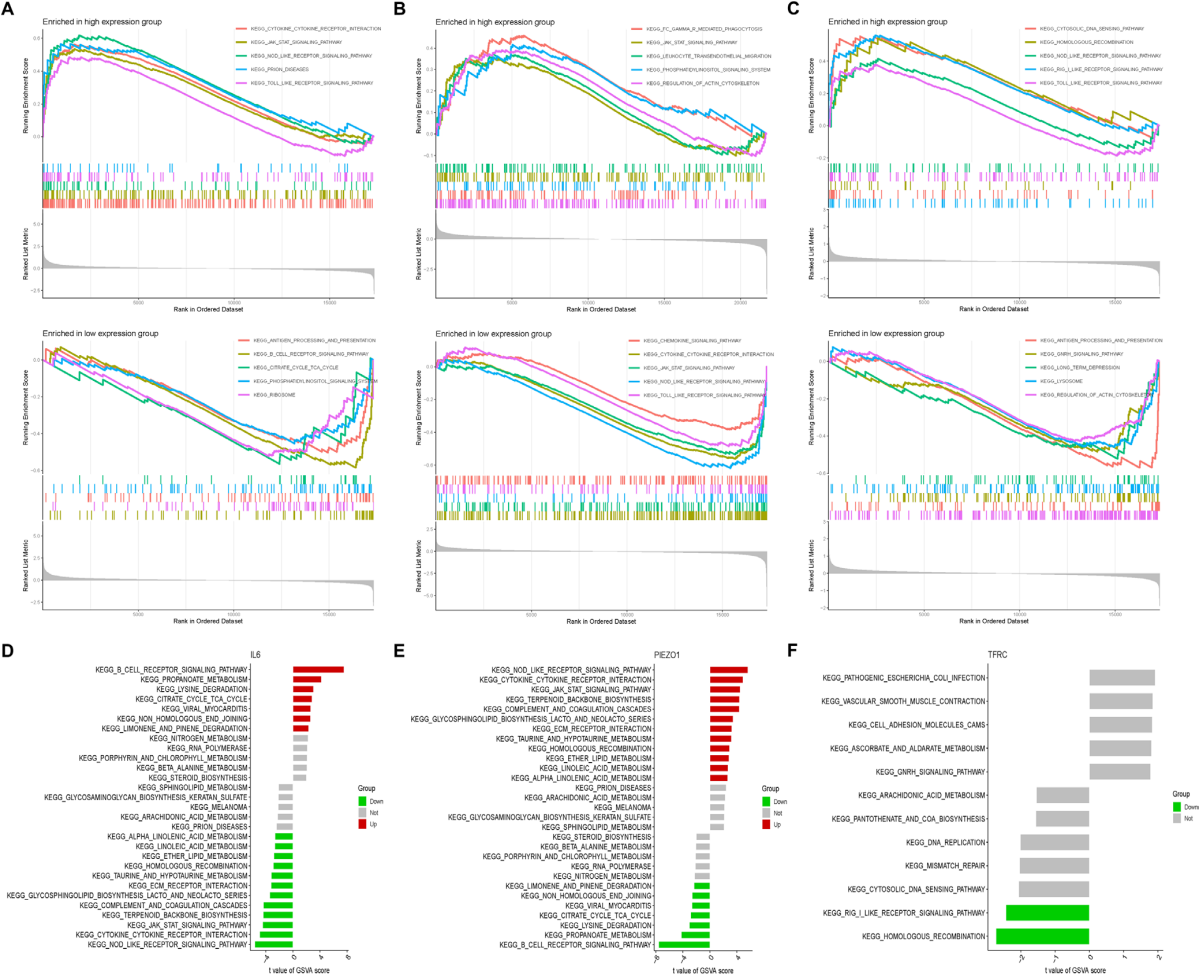
Function enrichment analysis based on the hub DEGs. (A–C) Enrichment plots from GSEA analysis in the high expression subgroups and low expression subgroups. (D–F) Histogram exhibited the results of GSVA analysis for hub DEGs.

To investigate the function of hub DEGs in BPD, we performed further GSEA and GSVA analyses. GSEA analysis delineated enrichment of cytokine receptor interaction in the IL6‐high subgroup (Figure 5A), regulation of actin cytoskeleton in the PIEZO1‐high subgroup (Figure 5B), homologous recombination in TFRC‐high subgroup (Figure 5C). Meanwhile, the results also implicated enrichment of antigen processing and presentation in the IL6‐low subgroup and the TFRC‐low subgroup (Figure 5A–C), and chemokine signalling pathway in the PIEZO1‐low subgroup (Figure 5B). Moreover, GSVA enrichment analysis implicated homologous recombination in the pathogenesis of IL6‐associated, PIEZO1‐associated and TFRC‐associated BPD (Figure 5D–F). These results provided us with hub DEGs‐related potential functional pathways for studying the process of BPD occurrence.

### 3.5 The Role of PIEZO1 and NETosis in BPD

**FIGURE 6:**
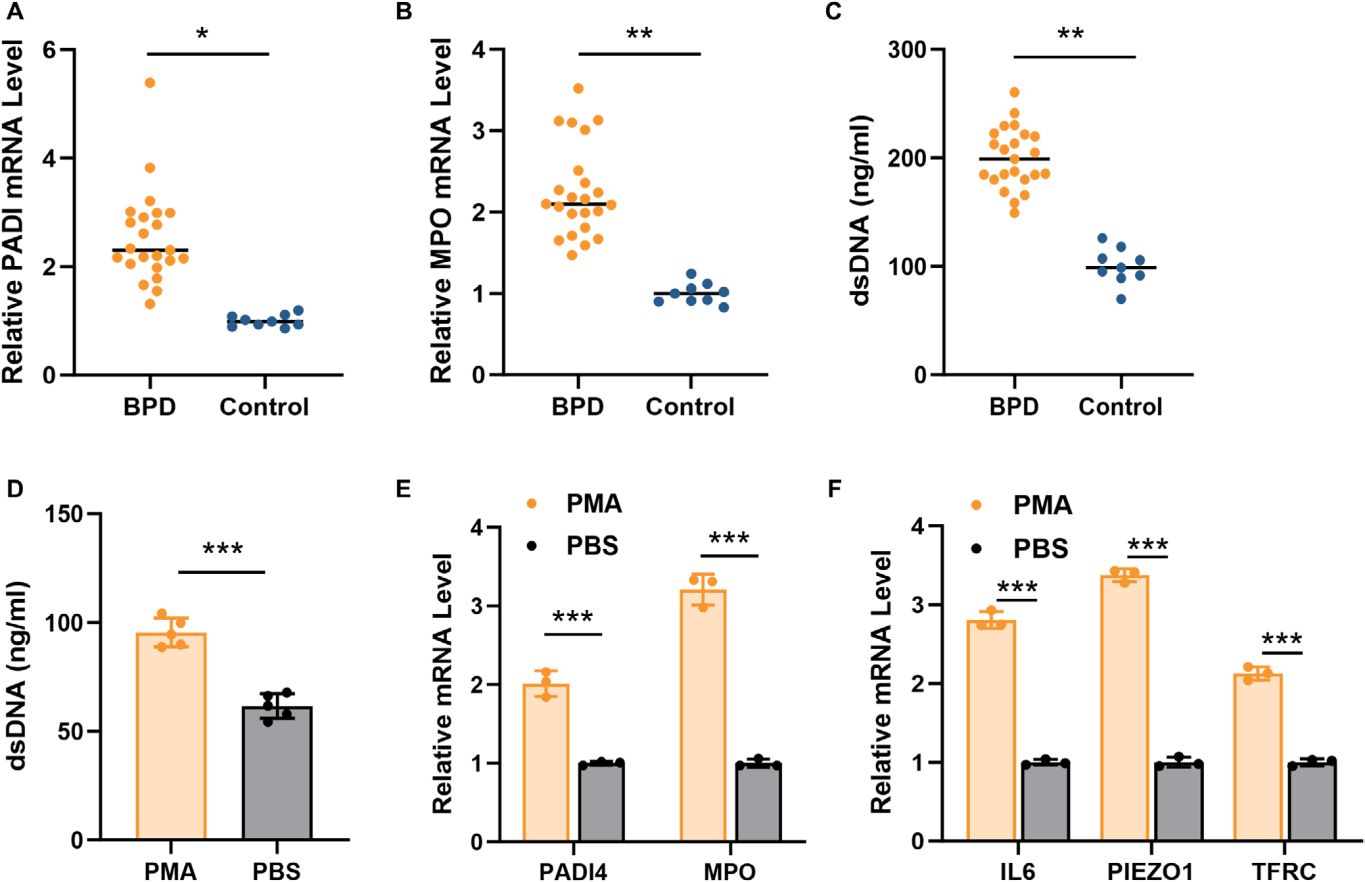
The role of PIEZO1 and NETosis in BPD. (A, B) PADI4 (A) and MPO (B) expression levels in BPD patients and healthy controls. (C) The serum dsDNA level in BPD patients and healthy controls. (D) The dsDNA level in supernatant from dHL‐60 cells treated with/without PMA. (E) PADI4 and MPO mRNA expression in dHL‐60 cells treated with/without PMA. (F) The expression levels of hub DEGs in dHL‐60 cells treated with/without PMA.

From August 2024 to March 2025, we obtained peripheral blood samples from 9 healthy controls and 23 patients with BPD diagnoses at Shanghai General Hospital. We measured the RNA expression levels of PADI4 and MPO in peripheral blood neutrophils from the samples in order to investigate the degree of NETosis in BPD. PADI4 and MPO expression levels were found to be much greater in the BPD group than in the healthy control group (Figure 6A,B). Meanwhile, the serum dsDNA level significantly increased in the BPD group (Figure 6C). According to these findings, the BPD group had a noticeably greater prevalence of NETosis.

PMA, a substance often used to cause NETs release in dHL‐60 cells, was given to the neutrophil‐like differentiated HL‐60 (dHL‐60) cells for four hours in order to examine the hub DEGs expression in NETosis. Figure 6D illustrates that cells treated with PMA had a greater quantity of dsDNA in the cell culture supernatant. The induction of NETosis was confirmed by the large increases in PADI4 and MPO mRNA levels (Figure 6E). Then, we detected the expression of three hub DEGs and found that all of them increased in cells treated with PMA (Figure 6F). Notably, the PIEZO1 gene showed the highest difference between the two groups among the three hub DEGs (Figure 6F). Further experiments consistently showed that the mRNA levels of PIEZO1 were significantly elevated in primary neutrophils from BPD patients compared to those from healthy controls (Figure S1). Hence, we chose PIEZO1 as the biomarker for NETosis in BPD.

### 3.6 PIEZO1 Promotes NETs Formation In Vitro via Regulating Calcium Homeostasis

**FIGURE 7:**
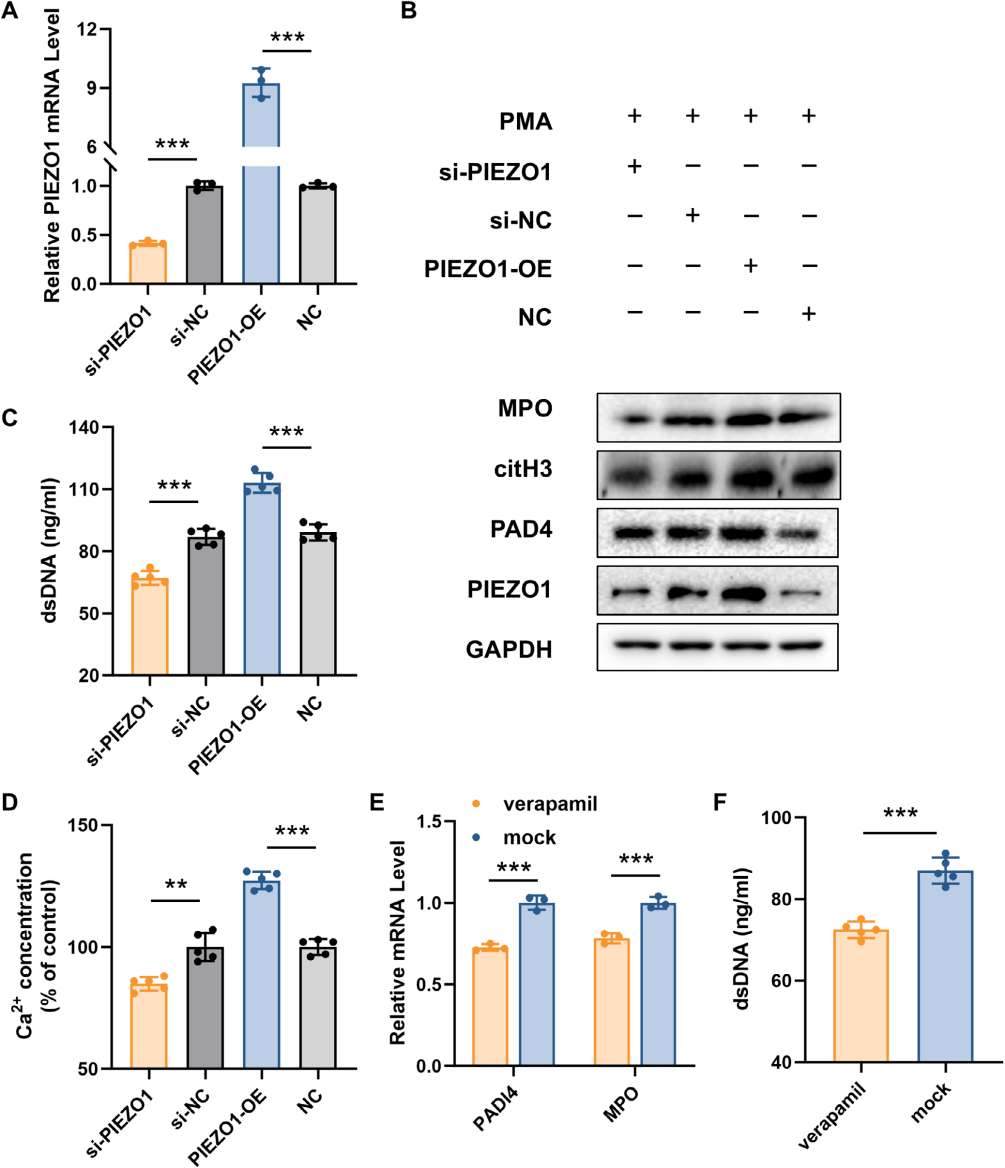
PIEZO1 promotes NETs formation in vitro via regulating calcium homeostasis. (A) The expression of PIEZO1 in dHL‐60 cells after the overexpression or knockdown treatment. (B) The protein levels of NET‐related proteins in dHL‐60 cells treated with PIEZO1 overexpression or knockdown. (C) The dsDNA level in supernatant from dHL‐60 cells treated with PIEZO1 overexpression or knockdown. (D) Intracellular Ca^2+^ concentration in dHL‐60 cells transfected with PIEZO1 knockdown or overexpression. (E) PADI4 and MPO expression levels in dHL‐60 treated with/without verapamil. (F) The dsDNA level in supernatant from dHL‐60 cells treated with/without verapamil.

To further evaluate the role of PIEZO1 in NETs formation in vitro study, we overexpressed PIEZO1 in dHL‐60 cells (Figure 7A). We observed that the mRNA level of NET‐related proteins (citH3, MPO and PAD4) was increased upon overexpression of PIEZO1 (Figure 7B), along with the increased dsDNA level (Figure 7C). In contrast, these NET markers were shown to be lower when siRNA suppressed PIEZO1 (Figure 7A–C). These results pointed to the idea that PIEZO1 overexpression sped up the development of NETs in neutrophils.

Prior research has shown that the release of NETs begins with surface receptor activation and is followed by intracellular calcium concentration changes [25]. Crucially, we observed a downward trend in si‐PIEZO1‐transfected cells relative to the control group, but a notable rise in cytosolic Ca^2+^ concentration in cells with PIEZO1‐overexpression (Figure 7D), suggesting that overexpression of PIEZO1 raises the intracellular calcium concentration in neutrophils. Then, using verapamil, a classical calcium‐channel blocker [26], we observed a reduction in PADI4 and MPO mRNA levels (Figure 7E) and dsDNA level (Figure 7F), indicating the decrease in PIEZO1‐induced NETs formation. These findings suggest that inhibition of the PIEZO1‐dependent calcium homeostasis alleviates NETs formation in neutrophils.

### 3.7 Overexpression of PIEZO1 in Neutrophils Exacerbates Pulmonary Fibrosis by Release of NETs

**FIGURE 8:**
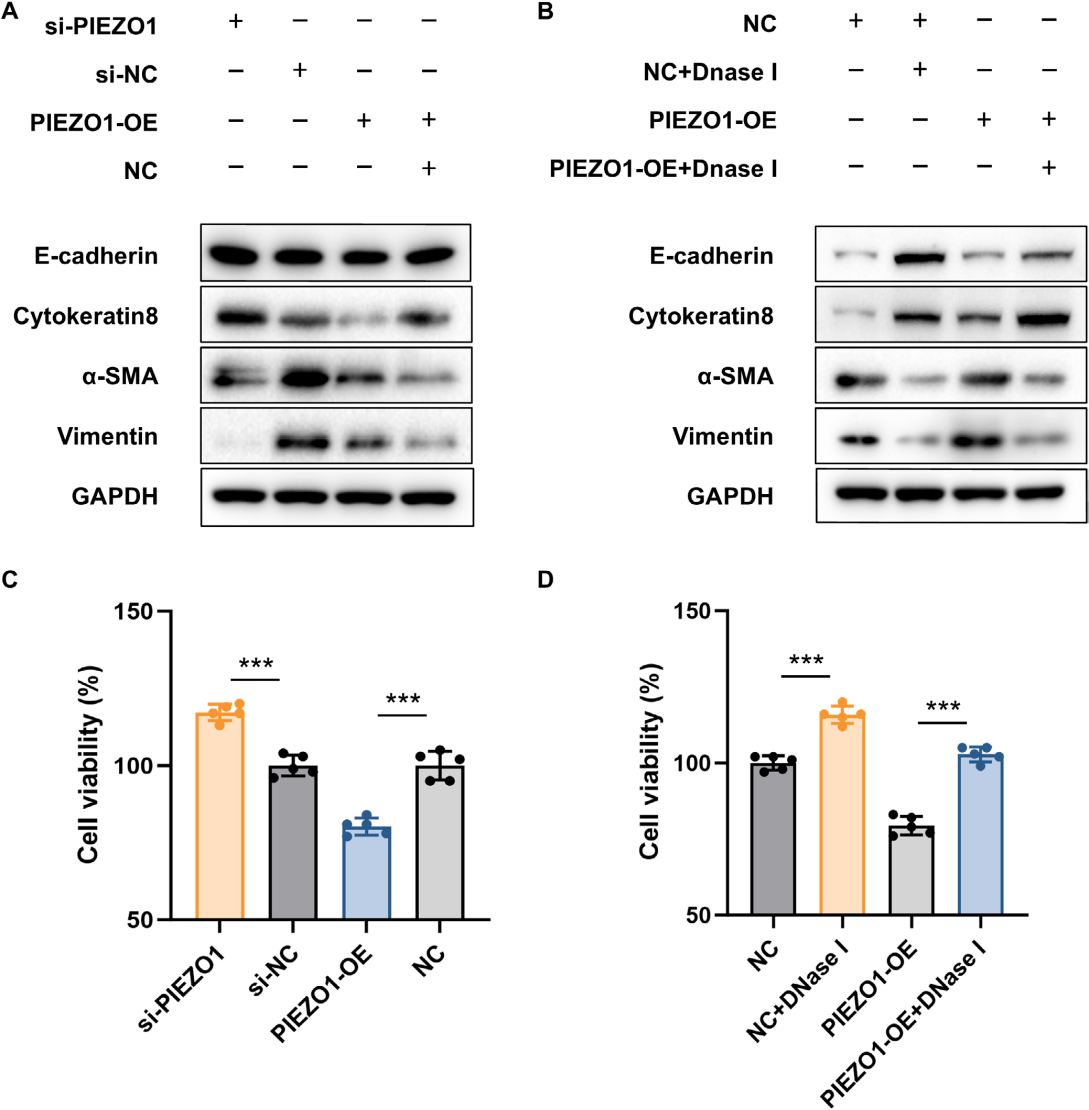
Overexpression of PIEZO1 in neutrophils exacerbates pulmonary fibrosis by the release of NETs. (A–C) The protein expression of EMT‐related genes (A) and cell viability (C) in the Beas‐2B cells, which were co‐cultured with dHL‐60 cells transfected with si‐PIEZO1 or pcDNA3.1‐PIEZO1. (B–D) The protein expression of EMT‐related genes (B) and cell viability (D) in the Beas‐2B cells, which were co‐cultured with PIEZO1‐overexpressed dHL‐60 cells treated with or without DNase I.

To investigate whether PIEZO1 expression in neutrophils controls pulmonary fibrosis by releasing NETs, we created a co‐culture system of dHL‐60 cells and Beas‐2B cells. The decrease and increase in PIEZO1 content in dHL‐60 neutrophils were achieved by siRNA transfection and plasmid overexpression, respectively. DNase I was added to dHL‐60 neutrophils to inhibit NETs formation. Western blotting demonstrated that fibrosis of Beas‐2B cells co‐cultured with PIEZO1‐low neutrophils was slightly reduced but significantly increased in PIEZO1‐high neutrophils compared with that of the control group (Figure 8A). Notably, the addition of DNase I reduced the level of fibrosis induced by co‐cultured PIEZO1‐high expression neutrophils to normal levels (Figure 8B). Further cell viability experiments (Figure 8C,D) confirmed the results. These findings suggest that inhibition of the PIEZO1‐dependent NETs alleviates the process of fibrosis in lung.

## 4 Discussion

In this study, we combined bioinformatic approaches with experimental validation to identify PIEZO1 as a novel regulator of NETs formation in BPD. Our findings point to a previously unrecognised mechanism in BPD through which this mechanically activated ion channel‐related gene contributes to inflammatory lung injury and interstitial fibrosis in the developing neonatal lung.

Through integrated analysis of multiple BPD‐related transcriptomic datasets, we identified three hub genes (IL6, TFRC and PIEZO1) strongly associated with the disease. Among these, PIEZO1 stood out due to its pronounced upregulation in BPD samples and its positive correlation with neutrophil infiltration. This observation is particularly intriguing given the established role of mechanical ventilation a major risk factor for BPD in inducing lung injury through excessive mechanical stretch [27]. PIEZO1, as a key mechanosensor, is well‐positioned to translate these physical forces into pro‐inflammatory signalling [28, 29]. Our results align with emerging evidence that mechanical stress can prime innate immune responses, although the specific involvement of PIEZO1 in neonatal lung disease has not been previously detailed.

Further analysis revealed a distinct immune landscape in BPD, marked by elevated levels of monocytes and neutrophils, alongside reduced regulatory T cells. The positive correlation between PIEZO1 expression and neutrophil abundance suggests that this channel may influence neutrophil activation or recruitment [30]. Importantly, in vitro experiments confirmed that PIEZO1 overexpression enhances NETosis, as indicated by increased expression of citrullinated histone H3 (citH3), MPO, PAD4 and elevated extracellular dsDNA. Conversely, PIEZO1 knockdown suppressed these effects. These data provide mechanistic support for the notion that mechanical activation of PIEZO1 exacerbates NET‐driven inflammation [31], a pathway likely highly relevant in ventilator‐induced lung injury.

A key finding was that PIEZO1‐mediated NETosis contributes directly to pulmonary fibrosis. Pulmonary fibrosis represents a critical pathological endpoint in severe BPD [32], characterised by excessive deposition of extracellular matrix proteins and disruption of normal alveolar architecture [33]. This fibroproliferative response is increasingly recognised as a major determinant of long‐term respiratory morbidity in BPD survivors [34]. Using a co‐culture system, we demonstrated that neutrophils overexpressing PIEZO1 induce fibrotic changes in lung epithelial cells (Beas‐2B), an effect abolished by DNase I treatment. This indicates that NETosis is a necessary intermediary in PIEZO1‐dependent fibrotic signalling. Previous studies have associated NETs with chronic lung pathologies, but our work is among the first to link PIEZO1 mechanotransduction directly to NET release and subsequent fibrosis in BPD. This finding is consistent with recent research showing that mechanical ventilation promotes PIEZO1‐mediated migration of airway smooth muscle cells, suggesting a broader role for PIEZO1 in ventilator‐induced lung injury [35].

The calcium‐dependent nature of PIEZO1 activation provides further insight into its mechanism of action. Our data show that PIEZO1 overexpression increases intracellular calcium concentration, while calcium channel blockade inhibits NETosis. This aligns with established literature on mechanosensitive channels [36] and extends to the specific context of NET formation, as demonstrated by studies showing that S100A12 triggers NETosis through calcium‐dependent pathways in myocardial infarction [37]. Although S100A12 was not the focus of the current study, the shared calcium‐mediated mechanism underscores the importance of ionic signaling in NETosis across different disease contexts.

It is important to take into account the many limitations of this research. First, our results may not be as broadly applicable as they may be due to the modest size of the clinical validation group. Second, because this was a preliminary investigation, we did not systematically account for confounding variables in every patient, such as concurrent medications or comorbidities (e.g., neonatal septicaemia and neonatal necrotising enterocolitis), which are known to affect immune cell function and systemic inflammation. Therefore, in order to control for these confounders and establish definitive clinical validity, the observed associations among PIEZO1, NETosis markers and BPD need to be confirmed in larger, multi‐centre prospective cohorts with thorough clinical data collection. In addition, our study's interpretation of the immune infiltration data is a significant shortcoming. Transcriptomic data from peripheral blood or cell models were used for the ssGSEA. Although this offers important information on the systemic immune state of BPD, it does not accurately represent the makeup of immune cells in the actual lung tissue. The systemic immune cell population changes that have been identified, such as the rise in neutrophils and monocytes, most likely indicate an elevated state of general inflammation that may either directly or indirectly lead to lung disease. Future research on lung biopsy samples using single‐cell RNA sequencing or spatial transcriptomics is necessary to confirm these results and accurately define the intra‐lung immunological landscape in BPD. Moreover, future research is needed to directly confirm the whole mechanistic route in primary neutrophils, even though our data reveals a pro‐NETotic function for PIEZO1 via calcium signalling in a differentiated cell‐line model and validates its high expression in primary neutrophils from BPD patients. Meanwhile, while the use of verapamil provides pharmacological evidence supporting the involvement of calcium signalling in PIEZO1‐driven NETosis, we acknowledge that future studies employing direct intracellular calcium chelators such as BAPTA‐AM would further strengthen the calcium‐dependency claim and help exclude potential off‐target effects. And, to completely define the pathophysiological influence of the PIEZO1‐NETosis axis on lung tissue damage, these results must be expanded utilising in vivo BPD models.

Overall, our results suggest that PIEZO1 is a viable and treatable target in BPD. An obvious next step is the creation of specific PIEZO1 inhibitors, such as small compounds or peptide‐based blockers like GsMTx4. These substances may selectively interfere with neutrophils' mechanotransduction‐driven NETosis, providing a focused treatment approach that might lessen lung damage without generally impairing immune function. Furthermore, our findings that PIEZO1‐induced NETosis is attenuated by the calcium channel blocker verapamil provide urgent therapeutic opportunities. It implies that a subgroup of BPD patients may benefit from the repurposing of currently available calcium‐signalling medications or the development of more targeted modulators of neutrophil calcium channels. Three areas will be the focus of our next work: to determine the effectiveness of treatment, these results must first be validated in preclinical in vivo models of BPD. Secondly, searching for particular PIEZO1 inhibitors in current chemical libraries. Thirdly, to develop future precision medicine studies that target this pathway, we will extend our clinical cohorts in order to verify PIEZO1 as a BPD biomarker. In conclusion, our study supports a model in which mechanical forces – such as those imposed by ventilation – activate PIEZO1 in neutrophils, promoting calcium influx and facilitating NET release. This process amplifies lung inflammation and fibrosis, ultimately impairing alveolarisation. Targeting PIEZO1 or its downstream NETosis effectors may offer novel therapeutic opportunities to mitigate lung injury in preterm infants. Future studies should explore the potential of selective PIEZO1 inhibitors or NETosis disruption as strategies to improve respiratory outcomes in BPD.

## Author Contributions

**Lei Cao:** conceptualisation, formal analysis, investigation, writing – Original Draft. **Yan Mao:** investigation, methodology, data curation, funding. **Chenxia Juan:** software, visualisation, funding. **Zhi Long:** validation, supervision, writing – review and editing. **Qian Wang:** conceptualisation, validation, writing – review and editing, funding. All authors read and approved the final manuscript.

## Funding

This work was supported by the Shanghai Municipal Health Commission Health Industry Clinical Research Special Project (202340060 to QW), the Natural Science Foundation of Shanghai Municipality (23ZR1451400 to QW), the National Natural Science Foundation of China (82400796 to YM, 82100753 to CJ), and the Excellent Young Doctoral Training Program of Jiangsu Province Hospital of Chinese Medicine (2024QB023 to YM).

## Ethics Statement

This study was conducted in compliance with the ethical principles of the Declaration of Helsinki and was approved by the Institutional Review Board (IRB) of Shanghai General Hospital. Written informed consent was obtained from all participants or their legal guardians for publication. The ethics approval number is 20240515062032022.

## Consent

The patients provided written informed consent for the publication.

## Conflicts of Interest

The authors declare no conflicts of interest.

## Supporting information


**Figure S1:** The difference in the PIEZO1 mRNA levels between BPD patients and healthy controls.

**Table S1:** The details of the NETs‐related genes obtained from the GeneCards database.

## Data Availability

The datasets presented in this study can be found in online repositories. The names of the repository/repositories and accession numbers can be found in the article. The data generated in the present study are included in the figures and/or tables of this article.
