# Supplementary figures and images for: Regulation of Calcium Homeostasis by PIEZO1 Drives NETosis and Fibrosis in Bronchopulmonary Dysplasia
Source: J Cell Mol Med. 2026 Mar 25;30(6):e71096. doi: 10.1111/jcmm.71096 (PMC13098034; doi:10.1111/jcmm.71096)

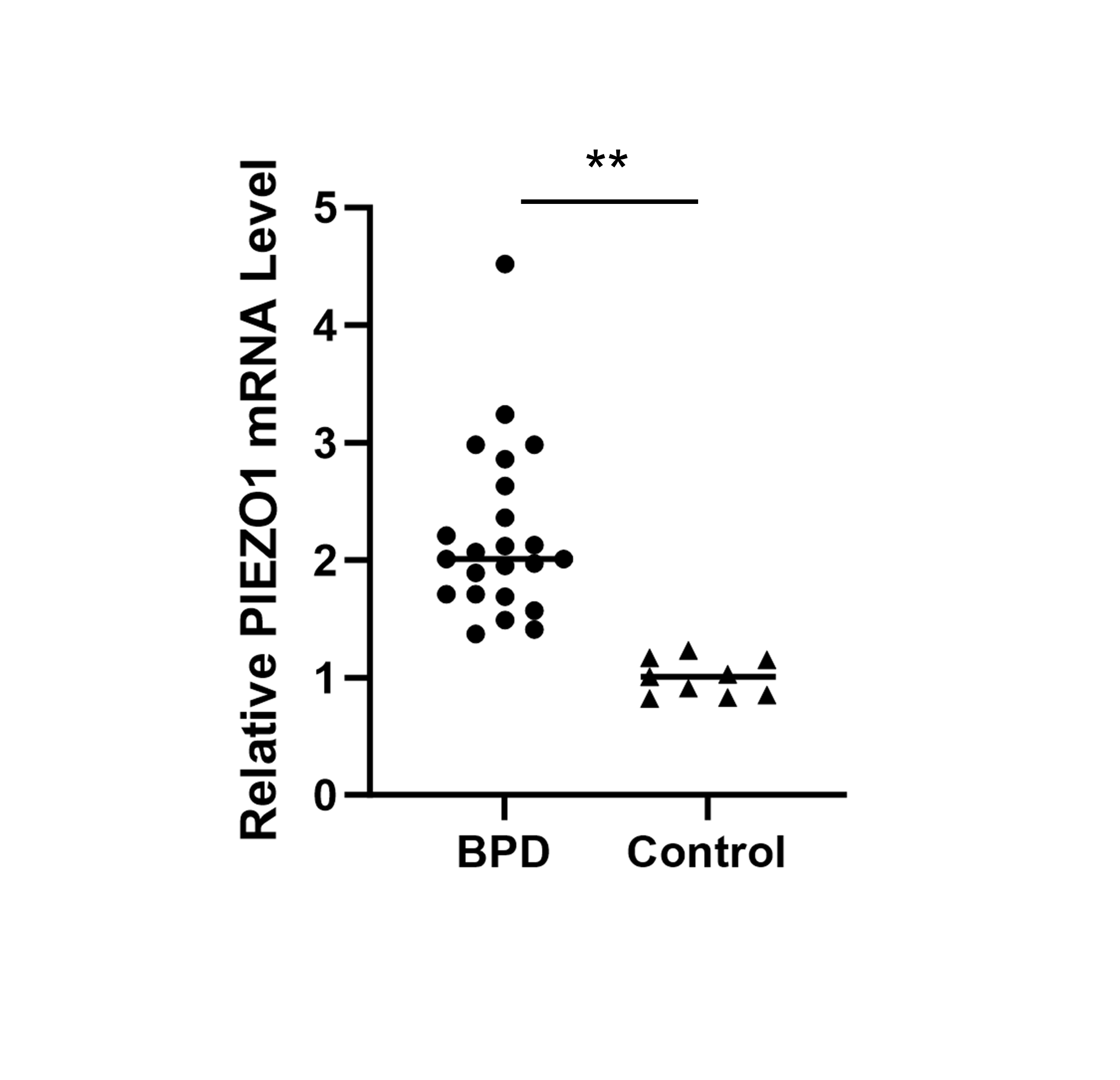

Supplement: Supplementary file 1 — Figure S1: The difference in the PIEZO1 mRNA levels between BPD patients and healthy controls. [file JCMM-30-e71096-s001.tif]
